# Challenges of sanitation in developing counties - Evidenced from a study of fourteen towns, Ethiopia

**DOI:** 10.1016/j.heliyon.2023.e12932

**Published:** 2023-01-20

**Authors:** Dagnachew Adugna

**Affiliations:** Ethiopian Institute of Architecture, Building Construction and City Development, Addis Ababa University, Ethiopia

**Keywords:** Developing countries, Ethiopia, Open defecation, Sanitation management, Water supply

## Abstract

Rapid urbanization and population growth in the past few decades has been worsening the water supply and sanitation problems in Ethiopia putting the current water supply deficit of the country at a staggering 41%. Using Ethiopia as a case of rapidly growing countries in the Global South and struggling with water supply and sanitation management, the objective of this study was to examine the challenges of sanitation in Ethiopia by selecting 14 towns located under different climatic conditions and administrative regions with diversified culture, ethnicity, and religion. Data from these towns were collected through household survey, Focus Group Discussion (FGD), Key Informant Interview (KII) and site visits. The field observation was conducted with representatives from the municipality who have knowledge on the existing sanitation and associated problems. Analysis of the collected data shows that poor water supply, inadequate toilet facilities, poor toilet facility emptying practices, poor community perceptions on sanitation management and inadequate emptying services were major challenges associated with sanitation. Moreover, absence of wastewater dumping site, lack of integration among the different components of sanitation, insufficient collaboration among potential stakeholders and gaps between the existing population and sanitation services were the other key challenges of sanitation exacerbated by inadequate financial resources. From the 14 studied towns the average water deficit was found 35%, and the average households with no toilet facilities were 17%. Only about 20% households have flushed toilets and about 5% practiced open defecation. While 42% of the households use vacuum trucks for emptying wastewaters and about 37% of the households dump wastewater outside of their premises. Among the studied 14 towns, only four towns have their own vacuum trucks, no town possesses wastewater dumping site. The different components of sanitation were managed separately without integration. Moreover, the collaboration among the potential stakeholders of sanitation management was found poor and fragmented. Also, sanitation services have not developed along with the population growth as the finance allocated to sanitation management is much lower compared to other municipal services. Thus, sanitation in the studied towns is poor, though there are progresses when compared with previous decades. To improve the sanitation condition in these towns the water supply should be improved together with raising the perception of the local community. The present study recommends further studies to be conducted on the feasibility of sustainable sanitation and “country-wide comprehensive” study on water supply, sanitation and open defecation in Ethiopian in particular and in developing countries as a whole.

## 1 Introduction

Access to improved water supply and sanitation is one of the basic needs and rights of every person. The health of the people and dignified life is ensured through access to improved water supply and basic sanitation. Improved water supply together with proper sanitation increases the health, social, and economic well-being of the people [1]. Ensuring access to improved water supply and basic sanitation services is the first step in eradicating poverty, especially in developing countries [2].

Despite urban areas are recognized as center for development, sanitation is one of the most critical problems facing it across the globe; . Cities contribute up to 5% of gross national product in low-income countries, 73% in middle-income countries and 85% in high-income countries [3]). Conversely, it is estimated that 668 million people globally lack access to improved water supplies and 2.4 billion people still live without access to improved sanitation [4]. There are significant disparities across regions, between urban and rural areas, and between the rich and the poor [5,6]. The disparities could be attributed to differences in economic growth, infrastructure development, awareness, housing investment, government, good governance and nongovernmental organizations interventions. Progress among the poorest is the slowest [7]. Furthermore, progresses witnessed during the United Nation Millennium Development Goals (MDGs) period disproportionately benefited the rich instead of the poor in most countries [8].The MDGs were aimed at reducing by half the proportion of people without access to safe drinking water and basic sanitation at the end of 2015. However, it missed the water and sanitation targets proposed in the MDGs [8]. Lack of access to improved drinking water is still a serious problem in developing countries where an estimated 675 million people have no access to improved drinking water [4]. Sub-Saharan Africa has the least developed sanitation infrastructure when compared to other developing nations. Compared to the global average of 36% without access to improved sanitation, 70% of the people in Sub-Saharan Africa use shared or poor quality sanitation facilities [9,10]. This implies that most of the households in Sub-Saharan Africa use “unprotected” and/or “non-networked” water supply sources [11,12].

To bridge the gaps of access to improved sanitation, significant financial resources, sustainable technological solutions that fit to the context of each local area and political determination are required.

Ethiopia, a sub-Saharan developing country, is the second most populous in Africa, with a population increase from 40 million in 1984 [13] to more than 110 million in 2020 [14]. Furthermore, a study conducted by [15] showed that Ethiopia is exhibiting a high annual rate of urbanization (5.4%). The Ethiopian urban population has more than doubled in the past 20 years, from 7.3 million in 1994 to 18 million in 2022 [14] with an annual growth rate of 6.82% between 2001 and 2019 [16], that is higher than the average in Sub-Saharan Africa (4.07%) [17]. However, the water supply and sanitation provision is not keeping up with the population growth. For instance, 39.74% of households in Ethiopia had limited access to drinking water services [18]. In contrast, [12] in its report revealed that water supply and sanitation facilities have been showing an increasing trend in Ethiopia, even though it still has the lowest water supply (42%) and sanitation coverage (28%) in sub-Saharan Africa. A study conducted by [19] showed that 52.1% of the Ethiopian population used unimproved sanitation facilities while 36% of them practiced open defecation.

Sanitation in Ethiopian urban areas is guided by the Sustainable Development Goals and the national sanitation management policies, but implemented only at an insignificant level with few traditional practices. This limited practice is the reason why significant proportion of the urban dwellers practice open defecation [20,21,22]. Therefore, the objective of this study was to investigate the challenges of sanitation in fourteen towns in Ethiopia located at different geographical locations with diversified culture, ethnicity, religion and socio-economic conditions.

Therefore, this study was conducted by surveying fourteen towns in Ethiopia so as to understand the state of water supply and sanitation, which together show the water supply and sanitation situation for the whole country. These towns were selected for a number of reasons among which rapid population and urbanization is one of them. The important challenge of the r growing population and urbanization is sanitation management. The rate of population growth in these fourteen studied towns for the period between 2007 & 2020 was 47%, with an average annual population growth rate of 4.29% (CSA, 2016). However, the provision of water supply and sanitation facilities were by far below the annual population growth.

The biggest challenge to proper sanitation in these towns may fall at the center of inadequate water supply, finance, aging infrastructure, population growth, urbanization, and climate change [23]. Moreover, the inability to link sanitation with other sanitation issues (e.g. solid waste, stormwater management), poor public perception, inadequate consideration of the social, cultural, political and economic factors into sanitation projects, the omission of potential actors, the assumption to implement a single technology that fits to all state of affairs, and the absence of multi-step process of sanitation from waste generation to disposal are the other key challenges. These result in overcrowding, slums, and squatter settlements in different parts of an urban area [24].

Using Ethiopia as a case representative of rapidly developing countries in the Global South, struggling with water supply and sanitation management, the goal of this study was to examine the challenges of sanitation, focusing on fourteen urban areas located under different climatic conditions. For fair representation the study focused at four settlements categories: slum, private residential houses, condominium houses and informal settlements, where little or no attention was given by previous studies [18,20,22,25] to such settlement combinations. This has created opportunities to reach most vulnerable and marginalized groups of the local community such as poorer households with variable socio-economic status, persons with disabilities, elders and women.

The results of this study will inform decision making on water supply and sanitation in developing countries in general and in Ethiopia in particular.

This paper is structured into six main sections: Introduction, Materials and Methods, Results, Discussion, Conclusions and Reference.

## 2 Materials and methods

### 2.1 Study area

**Figure 1:**
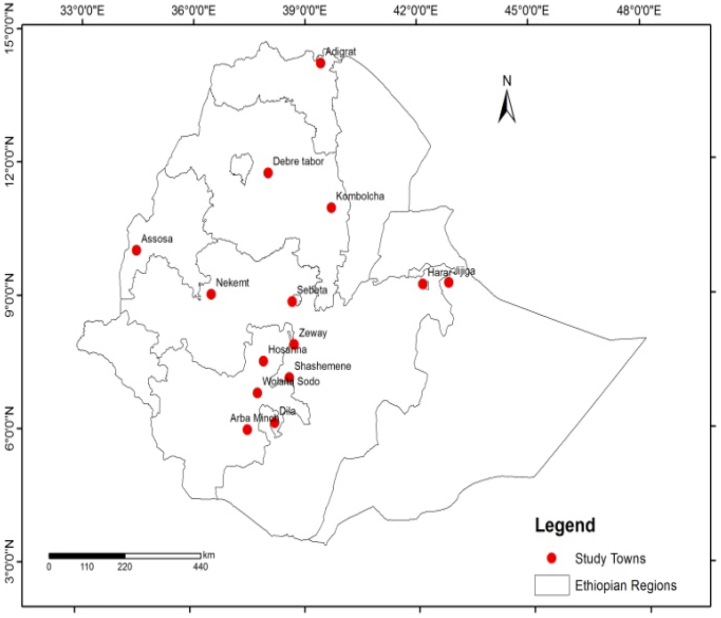
Location map of the study towns. *(source: own field survey, June to Sep. 2020)*

The study was conducted in fourteen towns in Ethiopia (Fig. 1). The fourteen towns were fairly distributed across Ethiopia within various climatic zones which could be representative to other towns of Ethiopia. The studied towns have populations greater than 50,000 which are classified as medium level urban areas in Ethiopia (Table 1).

**Table 1:** List of the study towns in Ethiopia with their geographical coordinate.

Towns	Geographical coordinates		Mean elevation, M
	Longitude, E	Latitude, N	
Arba Minch	37^0^29'32.83''	6^0^1'13.462''	1,225
WolaitaSodo	37^0^45'40''	6^0^51'20.48''	2,430
Hosanna	37^0^51'47.72''	7^0^33'47.654''	2,300
Zeway/Batu	38^0^5'1.76''	7^0^58'35.248''	1,643
Shashemene	38^0^35'43.17''	7^0^12'15.582''	2,200
Dila	38^0^18'37''	6^0^24'38''	1,420
Sebeta	38^0^39'26..95''	8^0^55'54.544''	2,300
DebreMarkos	37° 43' 47.82"	10° 20' 0.996"	2,425
Nekemt	36° 33' 19.39"	9° 5' 21.4836"	1,515
Asosa	34° 32' 3.73"	10° 3' 40.14"	1,513
Adigrat	39° 27' 38.02"	14° 16' 41.894"	2,457
Kombolcha	39° 44' 27.14"	11° 4' 52.889"	1,785
Harar	42° 7' 57.51"	9° 18' 16.73"	1,487
Jijiga	42° 47' 60.00"	9° 20' 60.00"	1,625

### 2.2 Data collection methods

This study employed mixed qualitative and quantitative methods. The data were gathered through households' survey, key informant interview, focus group discussion and personal observation from June to September 2020. During the qualitative and qualitative data collection, structured questionnaires were prepared and tested at a pilot town before starting the actual data collection.

#### 2.2.1 Households survey

Data related to water supply, wastewater collection, transportation and disposal mechanisms, sanitation management technologies, the impacts of poor sanitation at households, households perceptions towards sanitation management, coping strategies to management sanitation and challenges of sanitation management were collected through households survey. For the fourteen studied towns, the number of representative households was computed using the Cochran's formula (Eq. (2)). The formula was adjusted to the number of households for the towns at a response rate of 90% as all the questionnaires were administered by trained data collectors. First, a town level representative household sample size (n) was determined using Eq. (1).

(1) $$1n=\frac{z2\times p\times q}{\in 2}$$

After the value of "n" was obtained, the sample was adjusted to get the representaive households sample size, n_a_,using Eq. (2).

(2) $$\begin{array}{l} na=\frac{n}{\left[1+\left(n-1\right)/N\right]}\\ R \end{array}$$

Where;

*n*_*a =*_ sample households adjusted for response rate and total number of households
n = sample households required at Town level
$Z^{2}$= a standardized normal test with the consideration of α level of significance (the level of statistical significance at 95 percent confidence interval) (Z=1.96)
p = proportion value computed for each town by taking the proportion of urban households with improved toilet facility based on the 2016 DHS national urban estimate (0.16).
q = proportion of urban households with unimproved toilet facility (including open defecation), i.e. 1 - p (0.84).
Ɛ=level of precision required with a value of 5% (Ɛ=0.05)
*N* = total number of households in each town & *R*= response rate.

Accordingly, the calculated households size which were surveyed is presented in Table 2. Moreover, for fair representation and to consider the various contexts of the towns the household survey was conducted at four settlements categories: slum^1^, private residential houses, condominium houses^2^ and informal settlements^3^. The settlement categories were selected in discussion with the municipality. They were also selected to ensure the inclusiveness of the marginalized groups and settlement patterns in each studied town.

**Table 2:** Number of households participated in the households' survey.

Towns surveyed	Town population at the time of data collection^4^	Households surveyed					Annual mean rainfall (mm)
		Slum	Residential	Informal	Condo	sum	
Arba Minch	101,339	58	57	55	57	227	887.5
Wolaitasodo	102,923	58	58	55	57	228	1,212
Hosanna	94,728	57	57	57	56	227	1,196
Sebeta	60,834	56	58	56	56	226	1,020
Zeway/Batu	53,841	75	76	75	No	226	837
Shashemene	123,879	56	58	57	56	227	956
Dila	80,051	58	58	55	57	228	1,253
DebreMarkos	108,882	68	68	69	23	228	1,380
Kombolcha	102,244	68	67	67	25	227	1,038
Nekemt	121,385	57	57	57	56	227	1,998
Asosa	52,755	57	57	57	56	227	1,222
Adigrat	95,358	68	66	67	26	227	659.4
Harar	137,000	69	56	56	56	237	723
Jijiga	169,390	56	56	56	56	224	712

#### 2.2.2 Focus Group Discussion (FGD)

In addition to the households survey, a FGD was performed with a small group of people (as suggested by [26] which comprised of two individuals from influential elders, women's representative, youths' representative, health extension workers, environmental experts, water users forum representatives from the local community, local administration managers, local development representatives, school officers, water supply and sewerage enterprise experts, and sanitation and green development experts. The discussion was moderated by the researcher. Interview questions were presented to the FGD participants to get a detailed set of data about perceptions, thoughts, feelings and impressions of the local community and stakeholders in their own words [27]. In addition, questions were forwarded to the FGD participants to investigate the local community's understanding [28] related to sanitation management and associated challenges and to give opportunity to marginalized segments of society (e.g. elders, women, youths, extension workers) for exposing their feelings about sanitation and associated challenges.

#### 2.2.3 Key Informant Interview (KII)

A face-to-face KII was performed with main key stakeholders in each town which directly or indirectly involved in sanitation management. This included the town's water supply and sewerage enterprise, environment department, sanitation management and greenery development office, office of health, office of education, office of finance and development, department of infrastructure, department of urban planning and the city administration (or municipality). The KII was performed using structured questionnaires prepared ahead of time. The KII was planned to get data related to sanitation management and challenges, wastewater collection, transportation and disposal, water supply, and integration between the different components (e.g. water supply, wastewater, solid waste and stormwater management) of sanitation management. Furthermore, the environmental impacts of poor sanitation management practices, emptying services, collaboration among the different institutions involved in sanitation management, the availability of integrated sanitation master plan and the allocation of adequate finance for sanitation management were raised during KII.

The KII was executed to get the data indicated above from the limited number of well-connected and informed experts, to understand the perception and beliefs of the experts on the issues of sanitation management. Moreover, the KII was planned to acquire data from experts with diverse backgrounds and opinions and be able to ask in-depth and probing questions, to discuss at sensitive issues (e.g. major sanitation management challenges, disposal sites, collaboration among the stakeholders), to get an in-depth data related to the challenges of sanitation, and to create a comfortable environment where the experts can have a frank and open in-depth discussion.

#### 2.2.4 Field observation

Formal observations by the researcher with the help of representatives from the municipality who have knowledge on the different sanitation components and location of sanitation management facilities and problematic areas in each study town were made. The sites included the existing situation of representative sanitation problematic areas, public toilets, communal toilets, liquid waste dumping or disposal sites, schools, health facilities and drains.

### 2.3 Data quality assurance

To maintain the quality of the household data, the survey was conducted by nine graduates with first degree with a degree of civil, water supply, water resources, and hydraulics engineering. A half day intensive training was given to the field survey participants. They collected the data with close supervision by the researcher; and the day-to-day data were captured into a computer. The field survey participants were residents of the respective towns who are well-informed on the local language, tradition, religion, residents' way of life of each town.

## 3 Results

The major sanitation challenges identified from the fourteen studied towns are presented in following sections.

### 3.1 Water supply challenges

The annual water supply data as a key component of sanitation, collected from the corresponding water utilities of the 14 studied towns are presented in Table 3. The data in Table 3 indicates that the annual water supply of the studied towns as percentage of the annual water demand ranges between 46% and 85%. Correspondingly, the annual water supply deficit ranges between 15% and 64%.

**Table 3:** Water supply data and supply deficit of the fourteen surveyed towns.

Town	Annual water demand (m^3^)	Annual water supply (m^3^^5^)	Annual water supply as % of demand (%)	Deficit (%)
Arba Minch	2,475,000	2,123,725.5	85	15
Wolaitasodo	2,578,786	2,037,241	79	20
Hosanna	2,748,450	1,836,983	*67*	33
Sebeta	7,665,000	3,532,232	*46*	54
Zeway/Batu	1,652,070	983,028	60	40
Shashemene	5,033,350	3,419,991	68	32
Dila	1,782,000	1,228,000	71	29
DebreMarkos	3,487,327	1,186,682	50	50
Kombolcha	3,696,513	3,083,761	84	16
Nekemt	3,512,000	2,197,236	63	37
Asosa	1,347,890	1,000,200	74	26
Adigrat	2,715,600	983,759	36	64
Harar	3,615,544	2,581,971	71	29
Jijiga	3,165,000	1,859,125	59	41
Range			46 - 85	15 - 64
Average			67	43

Furthermore, the water supply sources of households both in normal circumstances and in times of water supply interruptions are shown in Table 4. The data revealed that from total surveyed households in the fourteen studied towns, households who got their water supply from protected sources (e.g. springs) range from 1% -22% and from unprotected sources (e.g. springs, rivers) range from 1%-14%, which needs intervention to correct it.

### 3.2 Poor and inadequate toilet facilities

I investigated that, of the total surveyed households from the fourteen studied towns, the majority of the surveyed households depends on dry on-site sanitation systems (66% - 94%), the remaining 34% - 6% depends on on-site water based sanitation system (i.e. flush toilet system) (Table 4). The detail results of the toilet facilities of these towns along with the various toilet categories and proportion of each use are presented in Table 5.

**Table 4:** Households water supply sources.

Town	Sources of water supply (%)		
	Tap water	Protected sources	Unprotected sources
Arba Minch	82	7	11
Wolaitasodo	88	2	10
Hosanna	83	8	9
Sebeta	91	2	7
Zeway/Batu	73	13	14
Shashemene	98	1	1
Dila	84	11	5
DebreMarkos	98.3	0.4	1.3
Kombolcha	79	16	5
Nekemt	86	13	1
Asosa	86	13	1
Adigrat	70	22	8
Harar	92	8	0
Jijiga	73	18	9
Range	*70 – 98%*	1 – 22%	1 – 14%
Average	*85%*	10%	6%

**Table 5:** Private toilet facilities and dependence of households with no toilet facility.

Town	Possess private toilets (%)	
	Yes	No
Arba Minch	78.4	21.6
Wolaitasodo	87	13
Hosanna	86	14
Sebeta	82	18
Zeway/Batu	84	16
Shashemene	74.4	25.6
Dila	92	8
DebreMarkos	94	6
Kombolcha	85	15
Nekemt	78	22
Asosa	78	22
Adigrat	74	26
Harar	88	12
Jijiga	72	28
***Range***	***72 - 94***	***8 -25.6***

Town	Flush	Improved	Unimproved	Communal	Public	Neighbors/relatives	Other	Open defecation
Arba Minch	33	30	37	6	12	30	50	2
Wolaitasodo	17	57	26	4	8	23	64	1
Hosanna	34	28	38	2	13	19	65	1
Sebeta	25	43	32	3	11	6	78	2
Zeway/Batu	11	51	38	2	3	23	71	1
Shashemene	13.7	39.6	47.7	4.4	4.4	10.6	77.6	3
Dila	14	51	34	3	3	13	80	1
DebreMarkos	14	33	53	4	8	4.5	74.8	8.7
Kombolcha	19	39	42	3.5	12	3.8	74.7	6
Nekemt	2	55	43	2	13	7	72	6
Asosa	2	40	58	6	9	9	70	6
Adigrat	2	54	44	4.2	14	11	62.3	8.5
Harar	16	49	35	2	17	16	62	3
Jijiga	11	63	26	6	16	17	54.5	6.5
*Range*	*2 -34*	*28 - 57*	*26 – 58*	*2 - 6*	*3 - 17*	*6 – 30*	*50-80*	*1 - 8.7*
*Average*	15.26	45.19	39.55	3.72	10.24	13.78	68.28	*3.98*

As shown in Table 5, of the total number of households surveyed from the fourteen towns who possessed toilet facilities such as flushed system, improved (with concrete slab and ventilation system) and unimproved (without concrete slab & ventilation system) ranges between 72% and 94%. Correspondingly, those households without toilet facilities range between 8% and 25.6%. Based on toilet facility classification, the same households survey revealed that the number of households who uses flush toilets ranges between 2% & 34%, improved toilet facilities between 28% & 57%, and unimproved toilet facilities ranges between 26% & 58%.

Similarly, I surveyed as where those households in the fourteen studied towns without toilets facilities commonly defecate and found out that 3% – 17% depended on public toilets, 2% to 6% used communal toilets, 6% to 30% used neighbors’ (or relatives’) toilets and 1% to 9% practiced open defecation. The number of households who did not want to disclose as where they defecate, due to private reasons, ranges 50% to 80%.

### 3.3 Poor toilet facility emptying practices

I also inspected the practices of households as what did they do when their toilet facilities get filled up. The result of the households' survey is shown in Table 6.

**Table 6:** Practices of households when their toilet facility gets filled up.

Town	Practices of households when their toilet facility gets filled up (%)			
	Emptying using vacuum trucks	Construct new toilet	Open defecation	Undefined
Arba Minch	43	30	2	25
Wolaitasodo	43	41	1	15
Hosanna	36	37	1	26
Sebeta	48	10	2	40
Zeway/Batu	37	40	1	22
Shashemene	33	30	3	34
Dila	44	41	1	14
DebreMarkos	36.3	35	8.7	20
Kombolcha	61	15	6	18
Nekemt	54	25	6	15
Asosa	29	31	6	34
Adigrat	36	22	8.5	33.5
Harar	65	17	3	15
Jijiga	58	17.5	6.5	18
*Range*	33 – 61%	10 – 41%	1 – 3%	14 – 34%
*Average*	45%	28%	4%	23%

As shown in Table 6, less than half of the households (average 45%) surveyed empty their toilet facility when filled up. The other 28% (average) construct a new toilet facility. The other 23% (average) were not willing to respond.

### 3.4 Poor community perceptions

The findings of this study revealed that the perception of the local community regarding wastewater management was found poor. This was verified by the results of the households (HHs) survey presented in Table 7.

**Table 7:** Households practice to manage grey wastewater.

Town	HHs dump wastewaters with their compound (%)				HHs dump wastewaters outside their compound (%)			HHs empty wastewaters to municipal dumping sites (%)^1^	Undefined (10%)
	open spaces	Soak pit	Septic tank	Sum	Splash outside	Stormwater drains	sum		
Arba Minch	16	23	21	60	10	20	30	21	10
Wolaitasodo	19	9	40	68	12	13	25	40	7
Hosanna	10	4	48	62	14	10	24	48	14
Sebeta	7	8	25	40	23	24	47	25	13
Zeway/Batu	15	8	15	38	33	17	50	15	12
Shashemene	10	12	22	44	33	11	44	22	12
Dila	13.6	4.8	15.8	34.2	28.1	30.3	58.4	15.8	7.4
DebreMarkos	13.3	39.3	24.7	77.3	14.3	6.7	21	24.7	1.7
Kombolcha	17.7	19.3	24	61	20.3	12	32.3	24	6.7
Nekemt	32.7	25.3	12.2	70.2	13.1	7.7	20.8	13.3	9
Asosa	32.3	25.3	13.3	70.9	10.3	7.7	18	13.3	11.1
Adigrat	23	4.3	24.7	52	5	3.7	8.7	24.7	39.3
Harar	11.3	34	14.7	60	21.7	13	34.7	14.7	5.3
Jijiga	24.7	20	19.7	64.4	19	9.7	28.7	19.7	7.9
Range				34-77			8.7-58.4		5.3-39.3

1 During households survey it was investigated that all wastewater in a septic tank dumped on to the open municipal liquid waste dumping site using either private or municipal vacuum trucks.

According to the results shown in Table 7, of the total households surveyed who dumped grey wastewater within their compound such as dumping on open spaces, soak pits and septic tanks ranges between 34% and 77%. The remaining 8.7% to 58.4% dumped outside of their compound either to stormwater drains or elsewhere. The other 5.3% to 39.3% of the households dumped to undefined places. From this, it is identified that the perception of the local community to manage grey wastewater either on-site or off-site was poor.

### 3.5 Inadequate or poor emptying services

The findings of this study showed that none of the fourteen studied towns possessed a sewerage system. They disposed wastewaters from septic tanks into an open dumping site using vacuum trucks. The households in these towns either use municipal or private vacuum trucks (Table 8) within their town or from other neighboring towns through leasing.

**Table 8:** Vacuum trucks and ownership in the fourteen studied towns.

Town	Ownership	
	Municipal	Private
Arba Minch	–	6
Wolaitasodo	1	1
Hosanna	–	3
Sebeta	1	3
Zeway/Batu	1	1
Shashemene	1	4
Dila	–	1
DebreMarkos	–	1
Kombolcha	1	3
Nekemt	–	1
Asosa	–	1
Adigrat	6	6
Harar	2	2
Jijiga	2	3

Table 8 revealed that of the 14 surveyed towns only 57% of them possess municipal vacuum truck. The remaining 43% depends on private vacuum trucks.

### 3.6 Absence of wastewater dumping site

**Figure 2:**
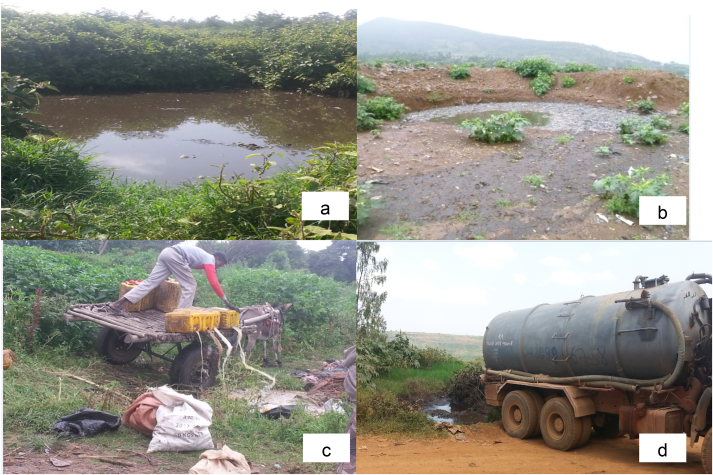
Poor open wastewater dumping sites; Hosanna (a), Sebeta (b), Arsi Negelle (c) & Dila (d) towns (Source: Captured by the researcher during data collection, June to Sep 2020).

The results of the present study revealed that none of the fourteen towns have a properly designed wastewater dumping site. All these towns dump the wastewaters by the side of solid waste dumping site. They dug a pit hole and dump the wastewaters there; they dug a new one when the other pit filled up. In almost all of the studied towns the pits overflow into downstream environment and contaminate water and land resources. In addition, some vacuum trucks dump wastewaters illegally somewhere else such as open spaces and agricultural fields. Some towns use simple donkey carts (Fig. 2) to transport wastewaters due to inadequate or absence of vacuum trucks. The management of wastewaters in the fourteen towns was found critically poor. As evidence, pictures captured during data collection are shown in Fig. 2.

### 3.7 Absence of integration among the different components of sanitation

**Figure 3:**
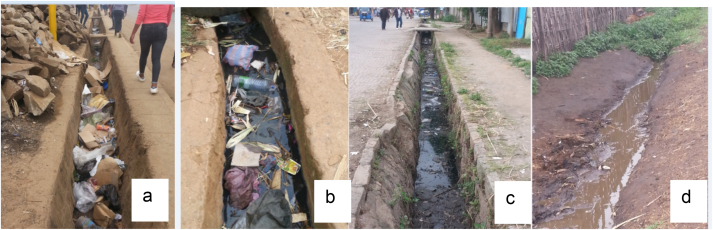
Dumping of wastes in to drains; Hosanna (a), Dila (b), Sashemene (c) & Arsi Negelle (d) towns (Source: Captured by the researcher during data collection, June to Sep 2020).

The present study discovered that the main components of sanitation such as wastewater, solid waste and stormwater were not managed through integration. These core elements of sanitation managed separately by fragmented institutions. Moreover, the towns’ master plan did not include a plan (or reserve spaces) for solid waste, and wastewater management. However, there is a stormwater management plan prepared separately for stormwater management. Generally, none of the fourteen towns had an integrated sanitation master plan to integrate wastewater, solid waste and stormwater management. This was signified that solid waste and wastewaters were excessively dumped into stormwater drains and elsewhere as shown in Fig. 3.

### 3.8 Poor or inadequate collaboration among potential stakeholders

The results of the present study revealed that the different potential stakeholders working with sanitation had poor or inadequate collaboration to manage sanitation through collaboration. The issue of water is managed by the towns' water utility, solid waste is managed by sanitation and greenery development unit, stormwater is managed by the department of infrastructure. The master plan department prepares the towns’ master plan without the participation of solid waste, wastewater and stormwater managing institutions. These stakeholders, in many cases, manage their activities separately through a fragmented approach, even if all of them are under same municipality. It was also found out that the towns' health office works with some elements of sanitation but still the issue of collaboration is poor or nonexistent.

### 3.9 Gap between the existing population and sanitation facilities

The existing sanitation facilities (e.g. public toilets, water supply, dumping sites) are inadequate which could not satisfy the present demand of the local community due to the fact that the population growth surpassed the sanitation demand of the current population. In fourteen of the studied towns the population growth is greater than the existing sanitation infrastructure, resulting in a gap between the existing sanitation infrastructure and the population. Most of the studied towns designed the water supply and sanitation infrastructure based on the normal forecasted population growth rate for a planning period of ten to fifteen years. However, in the middle of the designed period, the existing population surpassed the designed population by more than 25% due to the cumulative effects of migration and natural growth.

### 3.10 Inadequate financial resources

I investigated that the finance which was allocated for sanitation management in fourteen of the studied towns was inadequate. The surveyed towns also reported that there was no clear budget line for each of the sanitation management activities. Rather the budget is merged with other municipal services, though the water utility has its own budget line due its vast activities. The present study also revealed that for stormwater drains construction there is a capital budget allocated from external sources, nonetheless the focus is more on construction of drains than on stormwater management. Conventional drains are constructed every year because of the availability of funds from donors.

## 4 Discussion

### 4.1 Poor water supply

The average (85%) water supply to households in the studied towns is consistent with the study [20] conducted in ten Ethiopian urban areas. However, the same study reported that the water supply in Lalibela and Wolkite are 66% and 50% respectively, which are against the findings of the present study. This shows that Ethiopia failed to meet the MDG target, which is in agreement with the 2015 MDG assessment report that the improved drinking water in Sub-Saharan Africa is 42% [8]. Water is basic to sanitation [29]. However, the poor water supply in the majority of the studied towns complicated the situation of sanitation implying that some landlords closed the toilets for longer periods of time due to water shortage. Studies conducted in Sudan [30] and other Sub-Saharan Africa [31,32] are consistent with this study. Subsequently, users of toilets might have been forced to go on open defecation with greater probability for pathogens to be picked up by stormwater and join water resources. Moreover, residents fetching water from unprotected sources both during the time of water interruption and in normal circumstances might be infected by disease causing organisms [33]. This situation is more critical in high rise condominiums where the toilet facilities fully dependent on the municipal water supply system and the cumulative impact of these congested residents per a given block are worse than the situations seen in single residential settlements. Consistent with the present study, [24] reported that lack of constant water supply in many urban areas in the Sub-Saharan Africa is a major challenge of sanitation. Moreover, the findings of [34] agree with this study that municipalities have the responsibility to supply water to citizens.

### 4.2 Poor and inadequate toilet facilities

The majority of the households in the studied towns depend on dry on-site sanitation systems and some (5%, average) practiced open defecation (OD). Almost similar results were reported by [20] in their study of Ethiopia conducted at ten cities: Wolaita Sodo (5%), Wolkite (5%), Nekemt (5%), Kombolcha (5%), Adama (7%), Gondar (7%), Mekelle (7%), and Sebeta (8%), except Batu (10%) and Lalibela (20%). However, the average OD in the present studied towns is by far lower than in Kwale (52%) and Migori (33%) counties, Kenya [35]. A study conducted in Sudan by [36] reported that nationally 26% OD is practiced. A households survey [37] in Tanzania found out 40% OD. Generally, various studies [7,32,38,39] reported that open defecation is a common challenge in Sub-Saharan Africa. Moreover, the average OD in the studied towns is lower than the Ethiopian average OD (26.9%) [40] – this might be an indication that OD in the Ethiopian urban areas is declining from what it was thirteen years back. Consistent with this study, [41] reported that the Ethiopia’s annual rate of OD reduction exceeds that of more developed and well-resourced countries in sub-Saharan Africa. It is assumed that the declining of OD in Ethiopia could be directly associated with the active involvement of the paid health extension workers. Irrespective of the stated values, the existing practices of OD in the studied towns implying that the possibility of contaminating the local environment is high. This is because pathogens have higher potential to join water resources and contaminate agricultural fields and playgrounds [42]. Subsequently, children and local community depending on unprotected water sources are endangered to disease causing organisms such e-coli. The possibility of fly infestations on feces dropped around pit holes is far above the ground resulting in the spreading of communicable diseases such as diarrhea [43,44] from person to person. Such situations have implications on the residents' economy as they might frequent hospital visits for medication [37]. Moreover, the active workforce may be affected by diseases that reduce their effective working capacity/time leading to deprivation. In consistent with the present study, [45] reported that inadequate sanitation facilities caused by inadequate water supply, waste disposal, sanitation or drainage are the reasons of poor health. Furthermore, deprivations are bottlenecks to ensure sustainable urban social development as earnings of the poor goes to medication and treating the ill person. In agreement with this, studies conducted by [46] and [44] agreed that sustainable development is hardly possible where there is a high prevalence of unbearable illness and poverty, and the health of local community cannot be maintained without healthy environments and integral life-support systems.

### 4.3 Poor toilet facility emptying practices

Myers [47] underlined that fecal sludge management and toilet emptying are necessary for sustainable sanitation management. The lack of emptying dumping trucks forced most dwellers to construct new toilet facilities and reversion to OD when their toilet facilities filled-up, resulting in land and water pollution [39,48,49]. The OD practices in the studied towns could further result in a ‘slip’ back to OD as opposed to the Ethiopian target to end OD declaration by 2025 [50]. Moreover, the possibility of ‘slipping’ back to OD is against the Sustainable Development Goal Target 6.2. Poor empting and constructing new toilet facilities might also be the causes of communicable diseases and settlement of the land even after years of contamination. Studies conducted by [51,52] and [44] supported the present study that open defecation is the causes of many communicable diseases [53]. Open defecation could have a greater probability of polluting the nearby surface water resources [54] which then be consumed by those residents who depends on unprotected water sources and downstream community. Studies [44,51] revealed that open defecation is the causes of communicable diseases such as cholera, diarrhea [55] and dysentery that could be arisen from the release or discharge of e-coli, coliform into surface water bodies [21].

If constructing new pits or toilet facilities continue, the residents finish their holdings and re-dug the previous closed or abandoned pits which could further complicate the issue of sanitation and land contamination. Conversely, the abandoned pits might be the causes of soil collapse which could results in falling-in and death of children, elders and other members of the family, which is consistent with studies conducted in Uganda [32] and other 26 countries in Africa [44]. This has a negative implication on the family's economy either for health care or other means of medication. The findings of studies conducted in Ethiopia (Hunachew, 2016) and Kenya [37] are in agreement with this study. Thus, municipalities have the responsibility to introduce local-based sanitation management technologies which is consistent with studies conducted in Uganda [31] and Malawi [56]. This could array from waste generation to disposal and substitute such habitual and unsustainable practices. Dijk [57] revealed that sanitation management should focus on a multi-step process from waste generation to end use and must therefore be viewed in a value chain framework. This is in agreement with the present study.

### 4.4 Poor or weak collaboration among stakeholders

The collaboration among the institutions in the studied towns remains below expectation due to fragmented responsibilities, confusing institutional frameworks and lack of coordination among the multi-level fragmented governance arrangements. Studies [10,58] conducted in Uganda, Rwanda, Tanzania and Burundi are in agreement with the findings of this study. It should be recognized that urban sanitation management poses extraordinary challenges that cannot be solved by individual stakeholders. System failure in these towns is due to a top-down approach which limits involvement of stakeholders and the local community. Finding agreement on what the sanitation challenges are and how to solve them in these towns remains a big challenge, which is consistent with the study conducted by [23].

Another potential reason for system failure is the lack of understanding of the institutional setup in which the urban sanitation system is managed and operated. The methods and techniques developed were not appropriate for the local contexts, which is in agreement with the study conducted by [10]. Moreover, the social, political, cultural and economic factors were not taken into account, and there is no single institution that is best for all state of affairs of sanitation management. The lessons learnt from these experiences, has emphasized the need to recognize institutional arrangements and provide appropriate institutional developments and capacity building programs through well reinforced stakeholder collaboration. Consistent with this, studies [10,59] conducted in eleven Sub-Saharan and Asian countries underlined the need of capacity building and institutional collaboration. Moreover, a study conducted in Ethiopia, Ghana and Rwanda by [25] highlighted the need of cross-sector coordination and communication.

### 4.5 Gap between the existing population and sanitation services

In the studied towns, population growth and urbanization were found to be the most important challenges posed on sanitation management. Compatible with this, the report by the [3] revealed that the higher rate of population growth in developing countries complicates the management of sanitation.

It was investigated that population growth and rapid urbanization created a severe scarcity of water resulting in poor sanitation with substantial impact on the natural environment. This is in agreement with a study conducted by [60] in Sub-Sahara Africa that increasing the size of a household and an urban area decreases the likelihood of using improved water sources. Unless the towns meet their water demand either from ground or surface water sources the existing state of sanitation will further be worsened. Consistently, a study conducted in Sudan by [30] revealed that urban areas in developing countries have already been faced by massive backlogs in shelter, infrastructure and services and confronted with insufficient water supply, deteriorating sanitation and environmental pollution. The rise in population growth in the studied towns demands significant proportion of water to ensure sustainable sanitation which could decrease the burden of ecosystems to provide more regular and cleaner supplies.

Therefore, sustaining sanitation and achieving universal coverage in the studied towns represents a major challenge for human settlements, development and management. To bridge the existing gaps, flexible and innovative solutions are needed to deal with unexpected and significant changes in water demand for drinking and sanitation and associated economic activities [61,62,63].

### 4.6 Inadequate finance to complement the sanitation services

The absence of adequate finance to the sanitation sector holds back the development of sanitation infrastructure by the local community. Consequently significant number of the local community lacked the basic sanitation services. Studies [29,64,65] carried out in different parts of the world underlined the need of finance for sustainable sanitation. Moreover, the [50] in its integrated urban sanitation and hygiene strategy accentuated the necessity of financing the sanitation sector to promote sanitation services. However, as evidenced from the studied towns the local administrations paid lower interest on financing sanitation services. Conversely, the local community was fighting to meet their sanitation demand, though significant proportion of the local community was unlikely to meet the challenge of the sanitation needs. Thus, the local administration need to search for potential financers (e.g. Federal Government, development partners/NGOs, banks, micro-finances) to promote sustainable sanitation and reach the marginalized groups in the slum and informal settlements [44,66]; Apanga et al.; 2020; [65].

## 5 Conclusion

This study investigated the sanitation challenges in Ethiopia based on 14 representative selected towns across the country. The results of the study showed that the average water supply deficit across the 14 towns is found to be 35%, while Households (HHs) that do not have access to toilet facilities is 17%. It was also investigated that only 20% of the households have flushed toilets. The study further found out that 5% of the Households practiced open defecation and only 42% use vacuum trucks to empty their toilet facilities. Furthermore, nearly 37% of the HHs dump WW outside of their premises, only four towns have their own vacuum trucks. No town possessed WW dumping site. The foremost challenges identified in the studied towns are associated with a) poor water supply, b) poor and inadequate toilet facilities, c) poor and inadequate toilet emptying practices and services, d) poor community perceptions, and e) absence of WW dumping site. These challenges are attributed mainly to absence of integration among the different components of sanitation, fragmented governance arrangements, the inability of keeping up the sanitation services within the growing population due to inadequate financial resources and inadequate collaboration among potential institutions.

The observed sanitation challenges in the studied towns are principally associated with the higher population growth and rate of urbanization. Growing population together urbanization is increasingly creating gap in water supply and sanitation in both quantity and quality, particularly for dwellers in the condominium apartments, slum and informal settlements. Under dynamic increasing population, compared with declining water sources, access to water and sanitation will be decreasing with time. Associated water stress and health problems are extremely expected, particularly among dwellers in the slum and informal settlements. Moreover, it is likely that with limited resources and finances, development of improved water supply and sanitation services with a rate consistent to increasing rate of population and urbanization represents big challenge.

Moreover, the existing challenges happened due to dependence on a) short-term (reactive) measures instead of protective measures b) campaign instead of sustainable and long term measures c) donor-driven sanitation instead of demand-driven systems, d) ‘single’ approach instead of multiple or ‘context-based approaches, and focusing on constructing sanitation facilities before ensuring behavioral changes of the users.

The researcher is expected that ‘slipping’ back to OD may be happening due to the rise in cost of labor and construction materials. This could be more serious for the marginalized groups such as the poor, women, child and the elders. Moreover, the continuing unrest in different parts of the country may further complicate the sanitation challenges. However, working strongly on behavioral changes and culture in discussion with the local community and influential persons will at least help to maintain the lower status of OD when compared with other countries in Sub-Sahara Africa. Consequently, ‘slipping’ back to OD may be controlled and the Ethiopian ambition to declare open defecation free urban areas by 2025 and achieving the Sustainable Development Goals 6.2 may be ensured.

The author is of the opinion that this study will help policy makers and municipalities to develop sound sanitation management strategies and context-based sanitation management approaches as the different settlement categories need different interventions. This is because; there is no simple, single solution to all urban sanitation challenges, particularly in developing countries. It is recommended that locally relevant innovative sanitation solutions that put users first be implemented. Some of the limitation of this study are: 1) the absence of data on water quality testing of unprotected sources, 2) lack of original data on the impacts of open defecation on the general environment and associated epidemiological aspects that might be linked with unprotected water supply sources, 3) the assumption that the FGD and KII respondents could have reported what they thought the interviewer wished to hear (courtesy bias) and 4) inability to include the very updated data because of logistical and budgetary constraints, and security issues in some parts of the country. Thus, further comprehensive country-wide study on water supply and sanitation including the level of open defecation and its broad impact is recommended for urgent intervention.

## Author contribution statement

Dagnachew Adugna, Ph.D: Conceived and designed the experiments; Performed the experiments; Analyzed and interpreted the data; Contributed reagents, materials, analysis tools or data; Wrote the paper.

## Funding statement

This research did not receive any specific grant from funding agencies in the public, commercial, or not-for-profit sectors.

## Data availability statement

Data will be made available on request.

## Declaration of competing interest

The authors declare no conflict of interest.

## Footnotes

1 Slum refers to unplanned settlements with limited or no access to local roads, the per unit area per person is not to the standard. A number of scanty houses constructed one after the other without a border between the residences. It also generally lacks the basic social services.

2 Condominium houses refer to high rise building residential apartments constructed by the Ethiopian government & transferred to local community through lottery system. Each resident has its own toilet facility, in house piped water, and standard rooms.

3 Informal settlements refer to settlements without title deeds. Thus, they are with no access to legal water and electricity supply.

4 CSA, 2017.

5 m^3^ = cubic meter
